# Evidence of local adaptation to aridity but not nitrogen deposition in invasive annuals

**DOI:** 10.1002/ecy.70172

**Published:** 2025-07-29

**Authors:** Justin M. Valliere, Mayra J. Hernández, M. Rasoul Sharifi, Philip W. Rundel

**Affiliations:** ^1^ Department of Plant Sciences University of California Davis Davis California USA; ^2^ La Kretz Center for California Conservation Science University of California Los Angeles Los Angeles California USA; ^3^ Department of Ecology and Evolutionary Biology University of California Los Angeles Los Angeles California USA

**Keywords:** biological invasions, contemporary evolution, drought strategy, functional traits, genotype by environment interactions, global change, multivariate plasticity index

## Abstract

Rapid adaptation of traits and trait plasticity may contribute to plant invasiveness and confer fitness advantages in novel environments resulting from global change. The importance of trait differentiation in invasive plant populations is well recognized, particularly in response to climate. However, it is largely unknown how invasive plant populations will respond evolutionarily to atmospheric nitrogen (N) deposition resulting from air pollution, which is a major contributor to invasion success in many ecosystems. Using a common garden experiment, a widely used method for testing local adaptation, we assessed potential differences in plant functional traits and nitrogen plasticity across populations of two widespread invasive annuals from sites spanning a range of N deposition and aridity throughout southern California. These species exhibited clear population‐level differences in traits and N responses, but these were unrelated to N deposition. Instead, we detected significant relationships between several traits and aridity, and populations from more arid sites exhibited reduced N plasticity for multiple traits. Multivariate plasticity indices also showed a strong negative relationship with aridity across populations for both species. However, trait responses to N addition also appeared to be influenced by species' drought‐coping strategies. In *Bromus diandrus*, a drought‐escaping early‐season annual grass, populations from less arid sites showed increased plasticity in shoot growth and more rapid flowering in response to N addition. In contrast, *Centaurea melitensis*, a drought‐tolerant late‐season forb, showed climate‐driven shifts in biomass allocation in response to N; populations from more arid sites invested more in roots, while populations from less arid sites allocated more to leaves. These contrasting N responses strongly suggest distinct growth strategies and ecophysiological trade‐offs shaped by adaptation to local climate conditions. While elevated N availability may indeed promote invasion, climate stress might exert an overriding influence on local adaptation of plant invaders in dryland ecosystems subject to N deposition.

## INTRODUCTION

The role of climate in driving trait differentiation in invasive plant species is well recognized, with many studies reporting adaptive responses across climate gradients and in response to drought (Colautti & Barrett, 2013; Moran & Alexander, 2014; Nguyen et al., 2016). In contrast, surprisingly few studies have evaluated the evolutionary responses of plant species to atmospheric N deposition (Nguyen et al., 2016; Vergeer et al., 2008; Wedlich et al., 2016) despite the severe threat this poses to biodiversity (Bobbink et al., 2010). Elevated N deposition is a known driver of nonnative plant invasion (Davis et al., 2000; Fenn et al., 2010; Valliere et al., 2017) and may contribute to the expression of highly advantageous functional traits in invasives (Valliere, 2019; Valliere et al., 2022). Given the profound influence of N availability on competitive interactions and plant fitness, chronic N deposition might be expected to result in adaptive responses in plant invaders. For example, under high soil N, traits that improve a plant's ability to compete for light would increase fitness, resulting in selection for such traits (Tilman & Lehman, 2001). However, empirical evidence of such adaptive changes is extremely limited. In two studies from Europe, Vergeer et al. (2008) and Wedlich et al. (2016) reported population‐level differences in growth, phenology, and N use between plants from high and low N deposition sites. Conversely, Nguyen et al. (2016) found no evidence of evolutionary responses to experimental N deposition in two species of invasive grasses in California.

In addition to driving trait differentiation, environmental change may also exert selective pressures on phenotypic plasticity, which is itself a trait that may increase or decrease fitness (Matesanz et al., 2010; Van Kleunen & Fischer, 2005). The ability to adjust physiological, morphological, and phenological traits in response to a fluctuating environment can be an important contributor to invasion success, as it allows for the expression of advantageous phenotypes over a broad range of conditions (Matesanz et al., 2010; Richards et al., 2006). Such plasticity may have important evolutionary consequences under global change; if a greater capacity to express phenotypic variation confers greater fitness, this could result in adaptive plasticity in invasive populations (Matesanz et al., 2010; Moroney et al., 2013). However, greater plasticity may also be maladaptive in some contexts, and adaptation to other environmental factors may constrain plasticity (Valladares et al., 2007; Van Kleunen & Fischer, 2005). Thus, populations of invasive plant species that experience contrasting environments may undergo divergence in both traits and levels of trait plasticity. Whether or not increased phenotypic plasticity is positively selected for in populations will depend on environmental variability (Botero et al., 2015; Bradshaw, 1965). For example, in populations subject to highly variable interannual rainfall, greater plasticity would be expected to increase fitness and allow individuals to take advantage of favorable wet years. In contrast, if resource availability (e.g., soil N availability) is relatively constant in a given environment, greater plasticity would not confer such fitness benefits.

In this study, we sought to evaluate if two widespread invasive plant species in California, *Bromus diandrus* and *Centaurea melitensis*, exhibit population‐level trait differences in functional traits and plasticity to N addition and whether these responses are linked to site‐level climate conditions or N deposition rates. Post‐introduction changes in functional traits and increased phenotypic plasticity have been implicated in the invasion of *Centaurea* as well as other *Bromus* species in the western United States (Griffith et al., 2014; Moroney et al., 2013). In California, high levels of N deposition due to air pollution have also facilitated the invasion of ecosystems by nonnative annual grasses and forbs such as these (Cox et al., 2014), reducing plant diversity (Allen et al., 1996; Valliere et al., 2020) and elevating fire risk (Fenn et al., 2010). Yet, despite these severe ecological consequences, the potential role of local adaptation in driving invasion under N deposition is virtually unknown. Using a common garden experiment that included source populations from throughout southern California, we aimed to determine if these species exhibit population‐level differences in functional traits and N plasticity.

We hypothesized that (1) these species would exhibit population‐level differences in trait values that could be related to climate and N deposition. We also predicted (2) that populations from more arid sites would exhibit lower levels of phenotypic plasticity, since greater responsiveness to resource availability could be a riskier growth strategy in these more water‐limited environments. Finally, (3) while we expected some functional traits to exhibit population‐level differences in response to N, we predicted that levels of trait plasticity would be unrelated to N deposition rates experienced by source populations, as soil N availability at a given site is relatively constant.

## MATERIALS AND METHODS

### Study species

We selected two species native to the Mediterranean that are now widespread invasives throughout California (DiTomaso & Healy, 2007): *B. diandrus* and *C. melitensis*. *Bromus* is a winter annual grass that is self‐compatible and exhibits extremely low levels of outcrossing (Kon & Blacklow, 1990). *Centaurea* is a late‐season annual forb that produces both open (i.e., chasmogamous) flower heads with high levels of self‐pollination and closed (i.e., cleistogamous), obligately self‐pollinating flower heads (Porras & Muñoz Álvarez, 2000).

### Source populations

**FIGURE 1:**
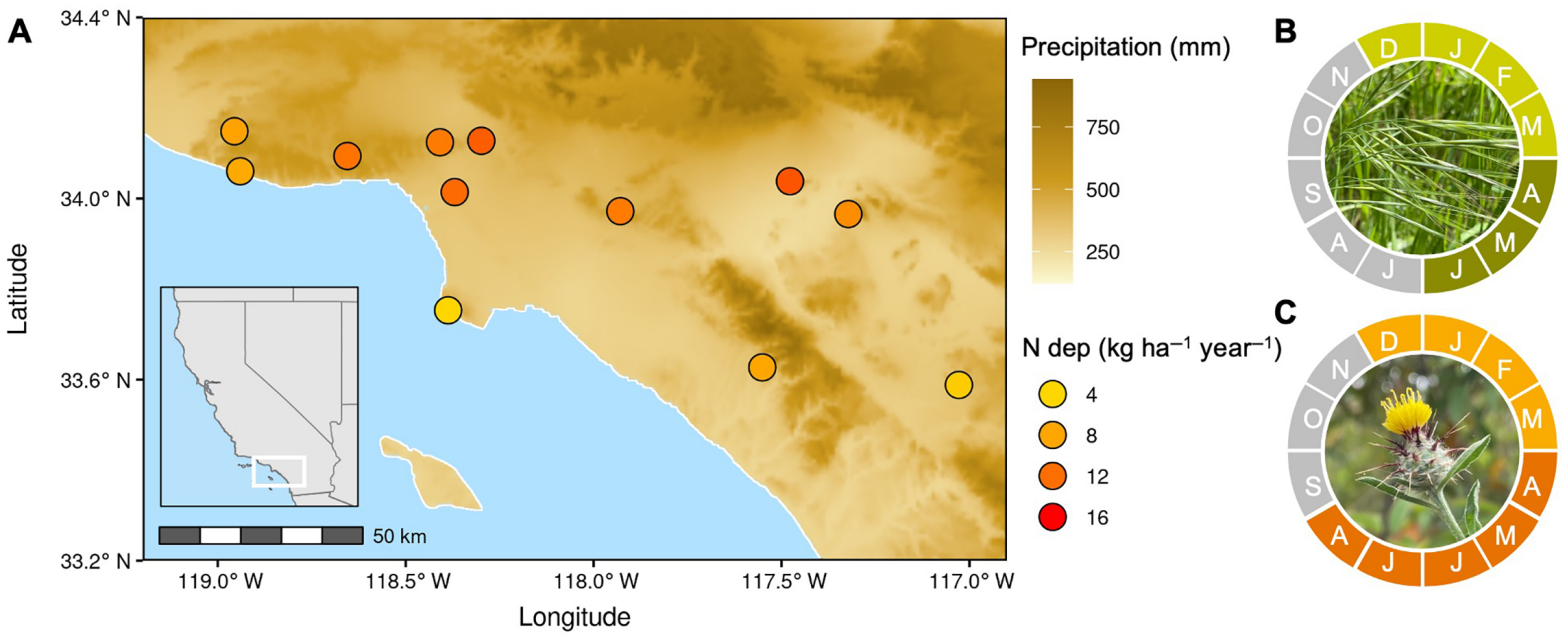
Locations of source populations of (A) *Bromus diandrus* and *Centaurea melitensis*. Shaded map area indicates average annual rainfall and circle color denotes N deposition rates. Phenology of (B) *B. diandrus* and (C) *C. melitensis* by month, with lighter and darker colors indicating vegetative growth and flowering, respectively. Months colored in gray are outside of a typical growing season. Photo credits: (B) B. Banfield and (C) Justin M. Valliere.

We collected seed of each species in 2017 from 12 study sites spanning a range of climate conditions and rates of N deposition (Figure 1; Appendix S1: Table S1). We initially selected sites based on N deposition values derived from the EPA's Community Multiscale Air Quality (CMAQ) model for 2002 (Fenn et al., 2010; Tonnesen et al., 2007). A more recent model was computed for 2002–2017 (Benish et al., 2022), and we used the average of values from this time period for data analysis. We acquired 30‐year averages for climate variables from the PRISM Climate Group at Oregon State University, USA (http://prism.oregonstate.edu). We used these data to calculate an aridity index similar to Welles and Funk (2021), which takes into account mean and maximum temperature, SD of temperature, precipitation of the wettest month, and coefficients of variation for precipitation (Harouna & Carlson, 1994). We selected this aridity index because it incorporates multiple climate variables and also enabled us to utilize reliable, site‐level climate data. In contrast, indices relying solely on precipitation and evapotranspiration were less suitable, as publicly available evapotranspiration data were coarser in spatial resolution and failed to accurately capture the variability in aridity across our study sites. This was particularly important given that some sites, despite their close proximity in southern California, experienced markedly different levels of aridity.

At each study site, we collected seed from 20 individual maternal plants within a one‐hectare area for each species. We avoided plants close to trails or roads and selected plants that were at minimum 2 m apart from other individuals from which seed was sourced. For *Centaurea*, we collected seeds from cleistogamous flower heads to limit the potential influence of gene flow. We weighed 20–30 seeds collected from each maternal plant and calculated mean seed mass.

### Common garden experiment

Plants were grown in a potted common garden experiment at the University of California, Los Angeles, from January to May 2018. Seeds were sown in conical pots measuring 3.8 cm in diameter and 21 cm in depth filled with potting soil consisting of a mix of sphagnum moss and perlite (Sunshine Mix number 4; Sun Gro Horticulture, Agawam, Massachusetts USA). Seeds were sown in 20 pots per population, with two pots for each population receiving seed from the same maternal plant. Pots were watered daily for the first week, and then 1–3 times per week as needed. Soil was allowed to dry out slightly between watering. We recorded the date of germination and thinned pots to a final density of one plant per pot after 2 weeks. After 5 weeks, we transplanted seedlings to round plastic pots measuring 22.6 cm in diameter and 21.6 cm in height. Following germination, one plant from each matched sibling pair was randomly assigned to a high or low N treatment. Our goal with these fertilization treatments was to simulate the high soil N availability that occurs early in the growing season at sites subject to elevated N deposition (Padgett et al., 1999). High N plants received three N fertilization treatments of ammonium nitrate (NH_4_NO_3_) in solution: once following germination, again after transplanting at 5 weeks, and a final time 2 weeks later. Low N plants received an equal amount of water each time. Each N treatment added approximately 20 μg N g^−1^ of soil. All *Bromus* plants survived the duration of the study (*n* = 10 per population and treatment combination; 240 replicate plants in total). For *Centaurea*, some plants died early in the experiment or failed to germinate, resulting in a slightly lower sample size (*n* = 8 per population and treatment combination; 192 replicate plants in total).

### Physiological measurements and leaf traits

We measured leaf gas exchange from randomly selected individuals (*n* = 4) in March 2018 (including photosynthetic rate, stomatal conductance, and transpiration) using a LI‐6400 portable open system (LI‐COR, Lincoln, Nebraska USA) equipped with a standard leaf chamber with LED light source and CO_2_ injector system. We measured recently mature leaves from each individual plant. All measurements were made on clear days during midmorning. Measurements were made at photosynthetically active radiation of 2000 μmol m^−2^ s^−1^, reference CO_2_ set to 400 μmol CO_2_ mol^−1^ air, and a flow rate of 500 μmol s^−1^. The temperature inside the chamber was kept close to maximum ambient temperatures. We made two measurements on each leaf and used the average of these two values for data analysis. Plant leaves were collected for subsequent measurements of leaf area in the laboratory using a LI‐1300 Leaf Area Meter (LI‐COR) to correct for values obtained from leaves that did not cover the entire leaf chamber. Leaf samples (*n* = 4) were dried in a forced air oven at 60°C for 48 h and weighed. We used these data to calculate specific leaf area (SLA; in milligrams per square centimeter). Dried leaf tissue was submitted for analysis of C and N content and δ^13^C at the UC Davis Stable Isotope Facility.

### Plant phenology and harvests

We recorded when each plant germinated and first exhibited mature flowers and used these data to calculate days to flowering (DTF). Because we were interested in evaluating differences in reproductive output, and plants matured at different rates, we harvested each plant when it first exhibited fully matured seeds rather than harvesting all plants at a single time point. Thus, our measures of plant growth reflect the total biomass and reproductive output these annual plants are able to attain before senescence. We separated shoots into leaves, stems, and reproductive biomass. Roots were washed with water to remove all soil. Plant biomass was dried for 72 h at 60°C before weighing. We calculated total biomass and root mass ratios (RMR; root mass divided by total plant mass) for each plant. We weighed a subset of mature seeds for each plant and calculated mean seed mass.

### Statistical analysis

All statistical analyses were completed in R (R Core Team, 2013) using RStudio (R version 4.1.0). Our aim was to evaluate population‐level differences in growth, phenology, and functional traits under high and low N availability, as well as differences in N plasticity. For each trait, we used two‐way ANOVA models with population, N treatment, and their interaction as fixed effects using the function “aov.” Data were transformed as needed to improve normality. To evaluate the potential influence of maternal effects on plant performance, we used ANOVA models to determine if mean seed mass of maternal plants from which seed was sourced differed by population. We then used linear models to test for the effect of population and N availability on total plant mass with mean seed mass of maternal plants included as a covariate. To compare levels of trait plasticity across populations, we calculated log N response ratios for each replicate grown under high N [ln RR = ln (trait value of high N plant/mean trait value of low N plants)]. We chose this measure of plasticity because it provides information on both the magnitude and the directionality (i.e., positive or negative) of N responses. We then used one‐way ANOVA models to evaluate the influence of source population on N responses. We then used linear regression to explore the influence of site‐level climate variables, aridity, and N deposition on mean trait values and N responses.

To evaluate potential differences in N plasticity across populations holistically, we used the multivariate plasticity index (MVPi) developed by Pennacchi et al. (2021). MVPi values were calculated for each population based on Euclidean distances between scores of a principal components analysis (PCA) of all plant traits for individuals grown under low and high N availability. This method provides an integrated measure of plasticity that takes into account changes in multiple traits simultaneously. We explored relationships between mean MVPi values for each population and aridity and N deposition using linear regression. We compared regression models that included just aridity to multiple regression models that included both aridity and N deposition using the “anova” function. The inclusion of N deposition did not improve model fits. We used PCA of scaled mean trait values using the “prcomp” function in the package *psych* to visualize differences in plant performance across populations and N treatments.

## RESULTS

Both species exhibited substantial population‐level differences in all measures of plant performance (Appendix S1: Tables S2 and S3, Figures S1–S4). Overall, N addition had a positive effect on plant growth, and most functional traits were significantly influenced by N availability (Appendix S1: Tables S2 and S3, Figures S1–S4). However, responses to N addition varied by population, as evidenced by a significant interaction between population identity and N treatment for many traits (Appendix S1: Tables S2 and S3) and a significant effect of source population on trait plasticity (Table 1). Populations differed in mean seed mass of maternal plants for *Bromus* (*F*_11,288_ = 10.06, *p* < 0.0001) and *Centaurea* (*F*_11,180_ = 4.34, *p* < 0.0001), but maternal seed mass had no significant effect when included as a covariate in linear models evaluating the effect of N and population identity on biomass for either *Bromus* (*F*_1,226_ = 1.01, *p* = 0.3165) or *Centaurea* (*F*_1,178_ = 2.08, *p* = 0.1515).

**TABLE 1:** Results of one‐way ANOVA models evaluating the effect of source population on the N responses (log response ratios) of plant growth metrics and functional traits for *Bromus diandrus* and *Centaurea melitensis*.

Trait	*B. diandrus*			*C. melitensis*		
	df	*F*	*p*	df	*F*	*p*
Root mass	11, 108	1.68	0.0876	**11, 84**	**14.10**	**<0.0001**
Shoot mass	**11, 108**	**5.40**	**<0.0001**	**11, 84**	**8.43**	**<0.0001**
Leaf mass	**11, 108**	**3.02**	**0.0015**	**11, 84**	**23.48**	**<0.0001**
Repr. mass	**11, 108**	**6.53**	**<0.0001**	**11, 84**	**3.65**	**0.0003**
Total mass	**11, 108**	**4.49**	**<0.0001**	**11, 84**	**5.24**	**<0.0001**
LMR	**11, 108**	**2.30**	**0.0142**	**11, 84**	**32.27**	**<0.0001**
RMR	**11, 108**	**1.91**	**0.0459**	**11, 84**	**20.95**	**<0.0001**
Seed mass	**11, 108**	**3.21**	**0.0008**	**11, 84**	**3.52**	**0.0004**
DTF	**11, 108**	**17.29**	**<0.0001**	**11, 84**	**3.68**	**0.0003**
*A*_max_	**11, 46**	**5.20**	**<0.0001**	**11, 36**	**4.68**	**0.0002**
*g*_*s*_	**11, 46**	**4.57**	**0.0001**	**11, 36**	**4.90**	**0.0001**
WUE	**11, 46**	**5.58**	**<0.0001**	**11, 36**	**9.02**	**<0.0001**
SLA	11, 46	1.78	0.0868	**11, 36**	**2.69**	**0.0123**
Leaf N	11, 35	1.23	0.3050	**11, 36**	**7.01**	**<0.0001**
Leaf CN	11, 35	1.69	0.1170	**11, 36**	**3.61**	**0.0017**
δ^13^C	11, 35	0.59	0.8280	**11, 36**	**3.73**	**0.0013**

*Note*: Values for significant models appear in boldface.

Abbreviations: DTF, days to flowering; LMR, leaf mass ratio; RMR, root mass ratio; SLA, specific leaf area; WUE, water use efficiency.

**FIGURE 2:**
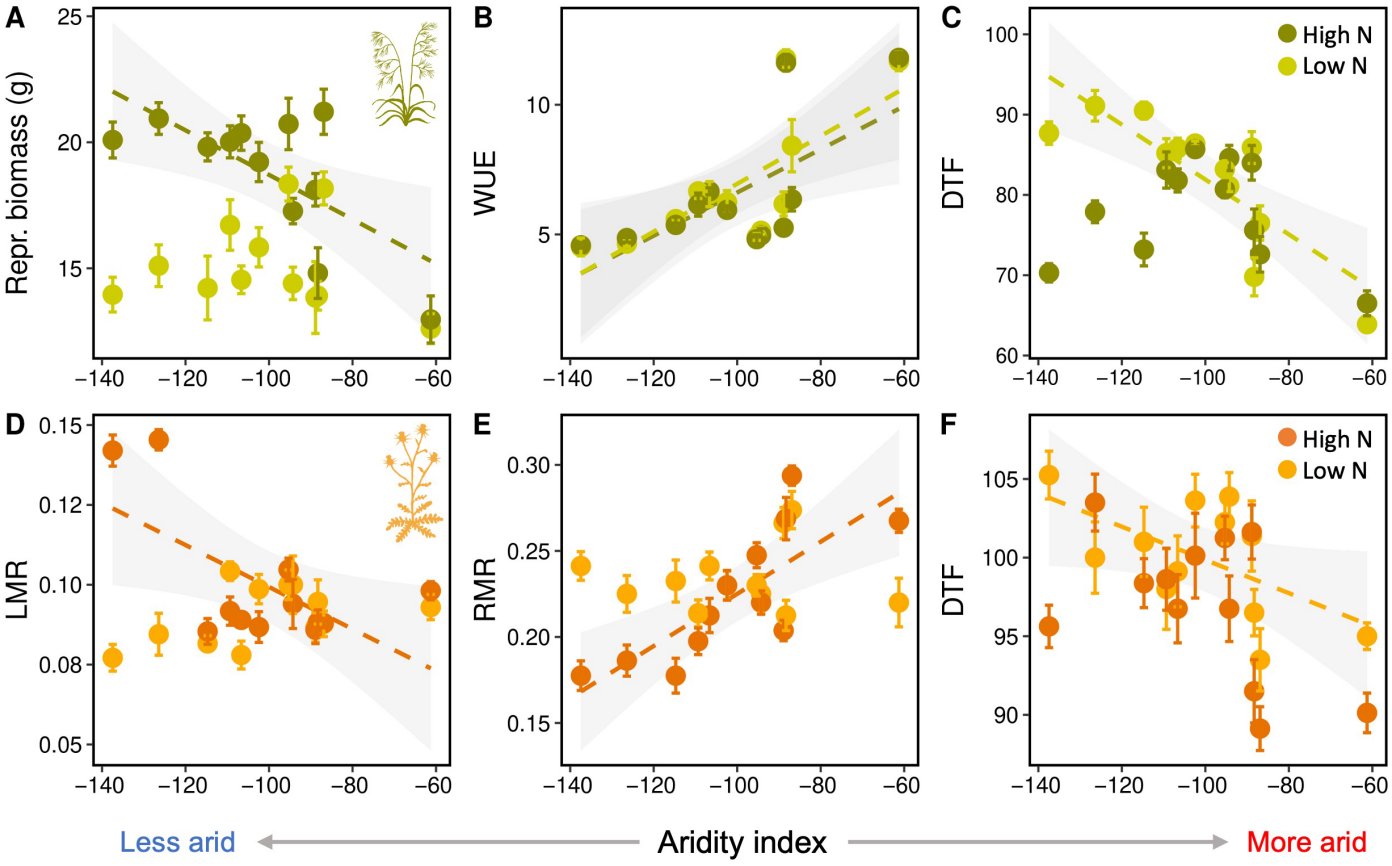
Linear regressions between aridity index and mean trait values across (A–C) *Bromus diandrus* and (D–F) *Centaurea melitensis* populations under both high and low N treatments, including relationships between aridity and (A) reproductive biomass, (B) instantaneous water use efficiency (WUE), and (C) days to flowering (DTF) for *Bromus* and (D) leaf mass ratio (LMR), (E) root mass ratio (RMR), and (F) DTF for *Centaurea*. Only significant regression lines (by N treatment) are shown along with 95% confidence bands in gray. Illustration credit: Justin M. Valliere.

Linear regressions revealed few relationships between mean trait values and site‐level environmental conditions across populations (Figure 2; Appendix S1: Table S4). Only a single trait showed a significant relationship with N deposition rates across source populations; in *Bromus*, photosynthetic capacity was negatively correlated with N deposition in plants grown under low N (*R* ^2^ = 0.33, *p* = 0.0304; Appendix S1: Table S4). However, this relationship was largely driven by high photosynthetic rates in a single population from the lowest N deposition site. Several traits in both study species were correlated with site aridity (Figure 2; Appendix S1: Table S4). In *Bromus*, reproductive mass was negatively correlated with aridity under high N (Figure 2A; *R* ^2^ = 0.42, *p* = 0.0140), with plants from more arid sites exhibiting lower reproductive output. Photosynthetic capacity was higher in *Bromus* populations from more arid sites under both low (*R* ^2^ = 0.33, *p* = 0.0306) and high N (*R* ^2^ = 0.35, *p* = 0.0246), and instantaneous water use efficiency (WUE) (Figure 2B) also increased across populations with increasing aridity under both low (*R* ^2^ = 0.49, *p* = 0.0065) and high N (*R* ^2^ = 0.39, *p* = 0.0180). In *Centaurea*, mean root biomass increased with aridity across populations under high N (*R* ^2^ = 0.58, *p* = 0.0026), while shoot (*R* ^2^ = 0.34, *p* = 0.0280) and leaf biomass (*R* ^2^ = 0.45, *p* = 0.0097) showed the opposite trend. These differences in allocation were also evident in regressions between leaf mass ratio (LMR) (Figure 2D) and RMR (Figure 2E) and aridity in *Centaurea*; under high N, LMR was higher in populations from less arid sites (*R* ^2^ = 0.33, *p* = 0.0307) while RMR increased with aridity (*R* ^2^ = 0.58, *p* = 0.0023) across populations.

*Bromus* flowered earlier than *Centaurea* (Appendix S1), but we detected similar relationships between flowering phenology and aridity in both species, as well as a similar impact of N addition on these relationships (Figure 2; Appendix S1). Under low N, populations from more arid sites flowered earlier than those from less arid sites in *Bromus* (Figure 2C; *R* ^2^ = 0.65, *p* = 0.0009) and *Centaurea* (Figure 2F; *R* ^2^ = 0.27, *p* = 0.0490). However, under high N availability, this relationship was absent for both *Bromus* (*R* ^2^ = 0.01, *p* = 0.7265) and *Centaurea* (*R* ^2^ = 0.19, *p* = 0.0881) due to more plastic responses in DTF in populations from less arid sites. When analyzed across N treatments, both *Bromus* (*R* ^2^ = 0.21, *p* = 0.0145) and *Centaurea* (*R* ^2^ = 0.22, *p* = 0.0121) showed a negative relationship between aridity and DTF, with more arid populations flowering earlier.

**FIGURE 3:**
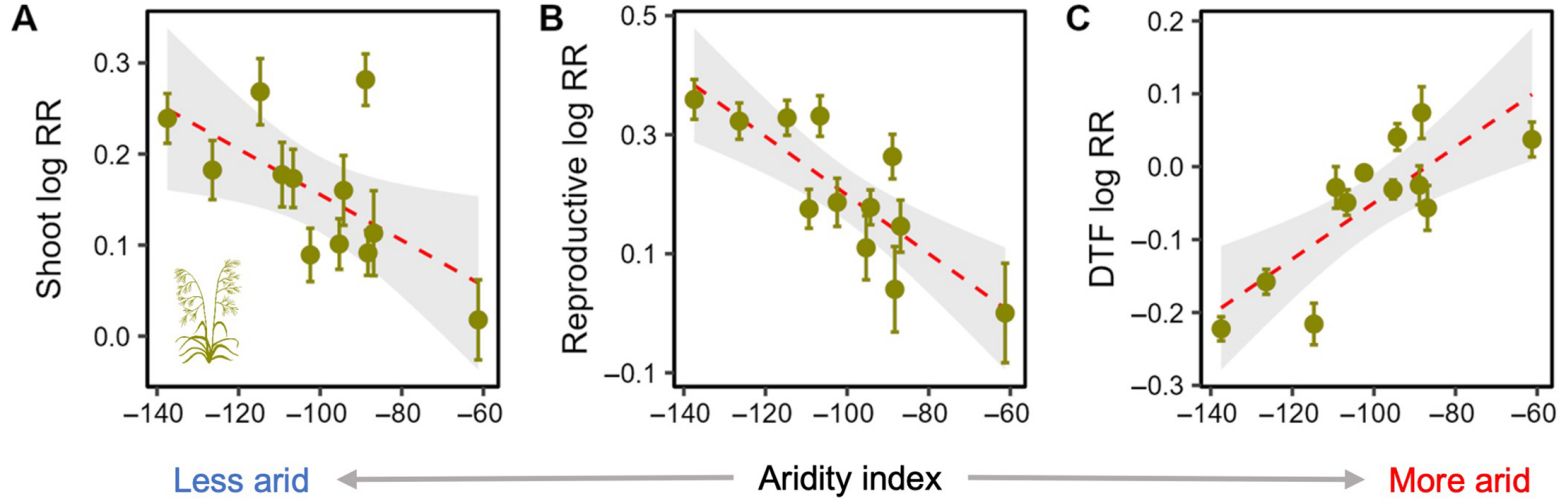
Linear regressions (and 95% confidence bands) between site aridity and log N response ratios (RR) across *Bromus* populations, including for shoot biomass, reproductive biomass, and days to flowering (DTF). Less negative aridity index values indicate more arid conditions. Illustration credit: Justin M. Valliere.

**FIGURE 4:**
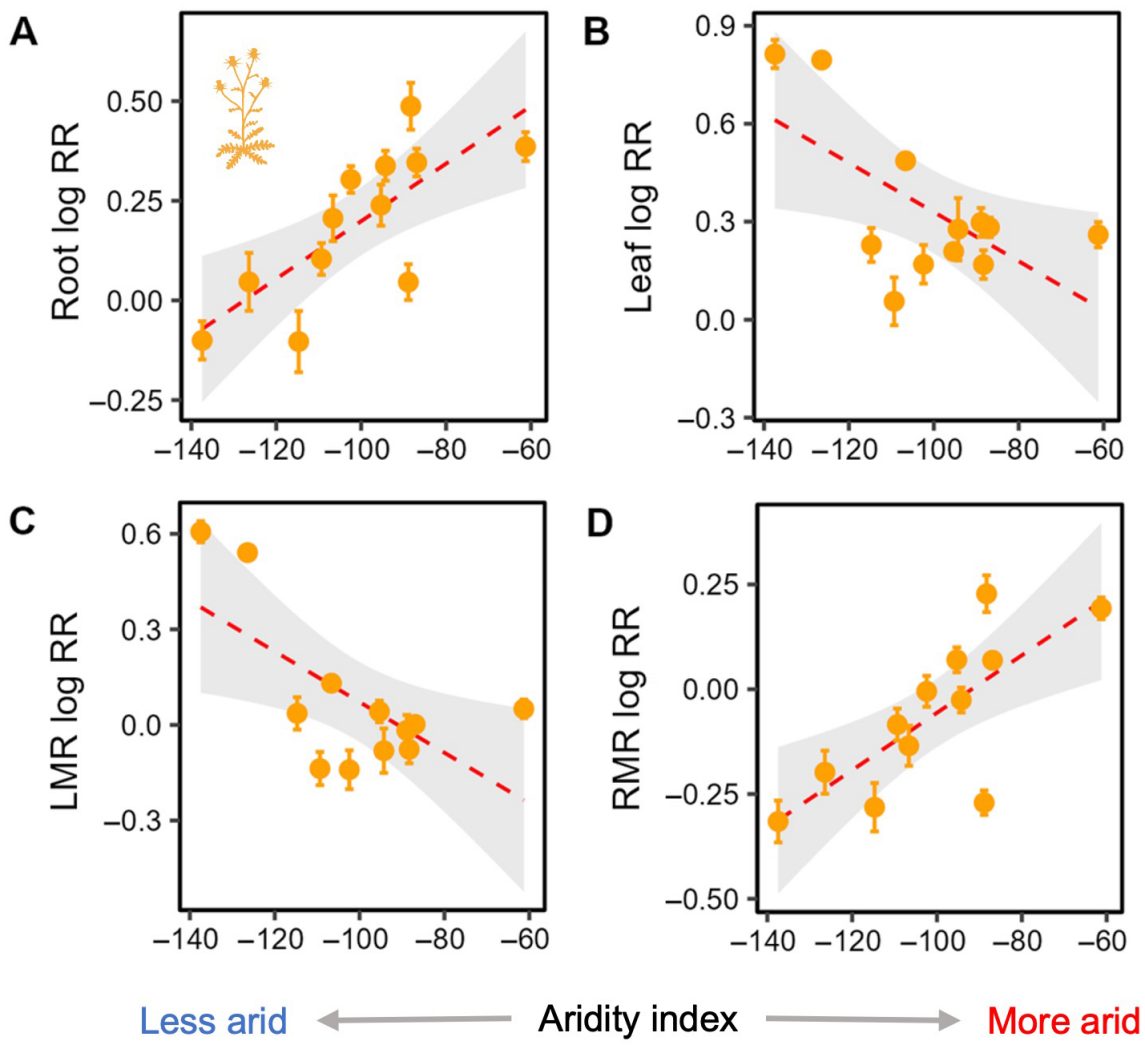
Linear regressions (and 95% confidence bands) between site aridity and log N response ratios (RR) across *Centaurea* populations, including for root biomass, leaf biomass, leaf mass ratio (LMR), and root mass ratio (RMR). Less negative aridity index values indicate more arid conditions. Illustration credit: Justin M. Valliere.

Populations showed significant differences in N plasticity for most traits (Table 1), and we examined how site‐level environmental factors influenced these responses (Table 2). We found no significant relationships between N plasticity and N deposition for any traits in either species; however, N plasticity for multiple traits was related to climate variables (Table 2). In *Bromus*, the plasticity of shoot and reproductive biomass was significantly affected by site aridity and precipitation, with populations from more arid sites exhibiting lower plasticity (Table 2, Figure 3). The response of *Bromus* populations' flowering phenology to N addition was also influenced by these climate variables (Table 2, Figure 3), with populations from more arid sites responding to N addition by flowering earlier. We also found a positive relationship between interannual rainfall variability and phenological plasticity in *Bromus* (Table 2). In *Centaurea*, the plastic responses of leaf and root biomass to N addition showed opposite relationships with site aridity (Table 2, Figure 4); populations from more arid sites exhibited more positive responses to N addition for root biomass, and populations from less arid sites responded to N addition with relatively larger increases in leaf biomass. These shifts in biomass allocation were further illustrated by regressions between LMR and RMR plasticity and aridity; populations from more arid sites showed greater plasticity for RMR but lower plasticity for LMR (Table 2, Figure 4). We also detected a negative relationship between N plasticity of shoot biomass and maximum temperature in *Centaurea*, with populations from hotter sites showing lower plasticity (Table 2).

**TABLE 2:** Coefficients of determination (adjusted *R* ^*2*^) from simple linear regressions evaluating the effect of N deposition, aridity, mean annual precipitation, variability in interannual precipitation (CV), and mean annual maximum temperatures on population‐level (*n* = 12) N responses (log response ratios) and multivariate plasticity indices (MVPi) for *Bromus diandrus* and *Centaurea melitensis*.

Trait	*B. diandrus*					*C. melitensis*				
	N dep	Aridity	Prec	CV	*T*_max_	N dep	Aridity	Prec	CV	*T*_max_
Root mass	0.07	−0.09	−0.08	−0.08	0.05	0.01	0.52**	0.52**	0.24	−0.09
Shoot mass	−0.10	0.34*	0.36*	0.03	0.04	−0.08	0.02	0.04	−0.05	0.50**
Leaf mass	0.21	−0.10	−0.10	−0.09	−0.10	−0.07	0.33*	0.35*	0.06	−0.10
Repr. mass	−0.03	0.66***	0.67***	0.13	0.03	−0.04	−0.06	−0.06	−0.10	0.23
Total mass	−0.07	0.19	0.19	−0.01	0.11	0.00	−0.08	−0.09	0.22	0.37*
LMR	0.18	−0.05	−0.06	−0.09	−0.05	−0.04	0.37*	0.37*	0.19	−0.07
RMR	0.05	0.16	0.17	0.03	−0.07	−0.06	0.52**	0.53**	0.07	0.04
Seed mass	−0.08	−0.03	−0.06	0.12	−0.10	0.09	−0.09	−0.09	−0.09	−0.10
DTF	0.26	0.60**	0.56*	0.38*	−0.07	−0.09	−0.09	−0.10	−0.01	−0.10
*A*_max_	0.25	0.17	0.18	0.01	0.17	−0.10	−0.09	−0.07	0.09	−0.10
*g*_*s*_	0.22	−0.01	−0.01	−0.08	0.19	−0.07	−0.10	−0.08	−0.03	0.00
WUE	−0.08	0.01	0.09	0.06	−0.10	−0.01	−0.04	−0.05	0.05	0.00
SLA	−0.10	−0.07	−0.09	−0.09	−0.10	0.05	0.16	0.12	0.20	0.10
Leaf N	−0.01	−0.08	−0.05	0.10	0.13	−0.09	−0.09	−0.09	0.11	−0.07
Leaf CN	−0.10	0.00	0.04	0.12	0.21	−0.10	−0.10	−0.09	0.07	−0.09
δ^13^C	0.18	0.15	0.14	−0.09	−0.07	−0.10	−0.10	−0.10	0.08	−0.06
MVPi	−0.07	0.47**	0.34*	0.39*	−0.10	−0.09	0.47**	0.44*	0.14	−0.01

*Note*: Asterisks denote levels of significance (*p < 0.05; **p < 0.01; ***p < 0.001), with significant regressions shown in boldface.

Abbreviations: DTF, days to flowering; LMR, leaf mass ratio; RMR, root mass ratio; SLA, specific leaf area; WUE, water use efficiency.

**FIGURE 5:**
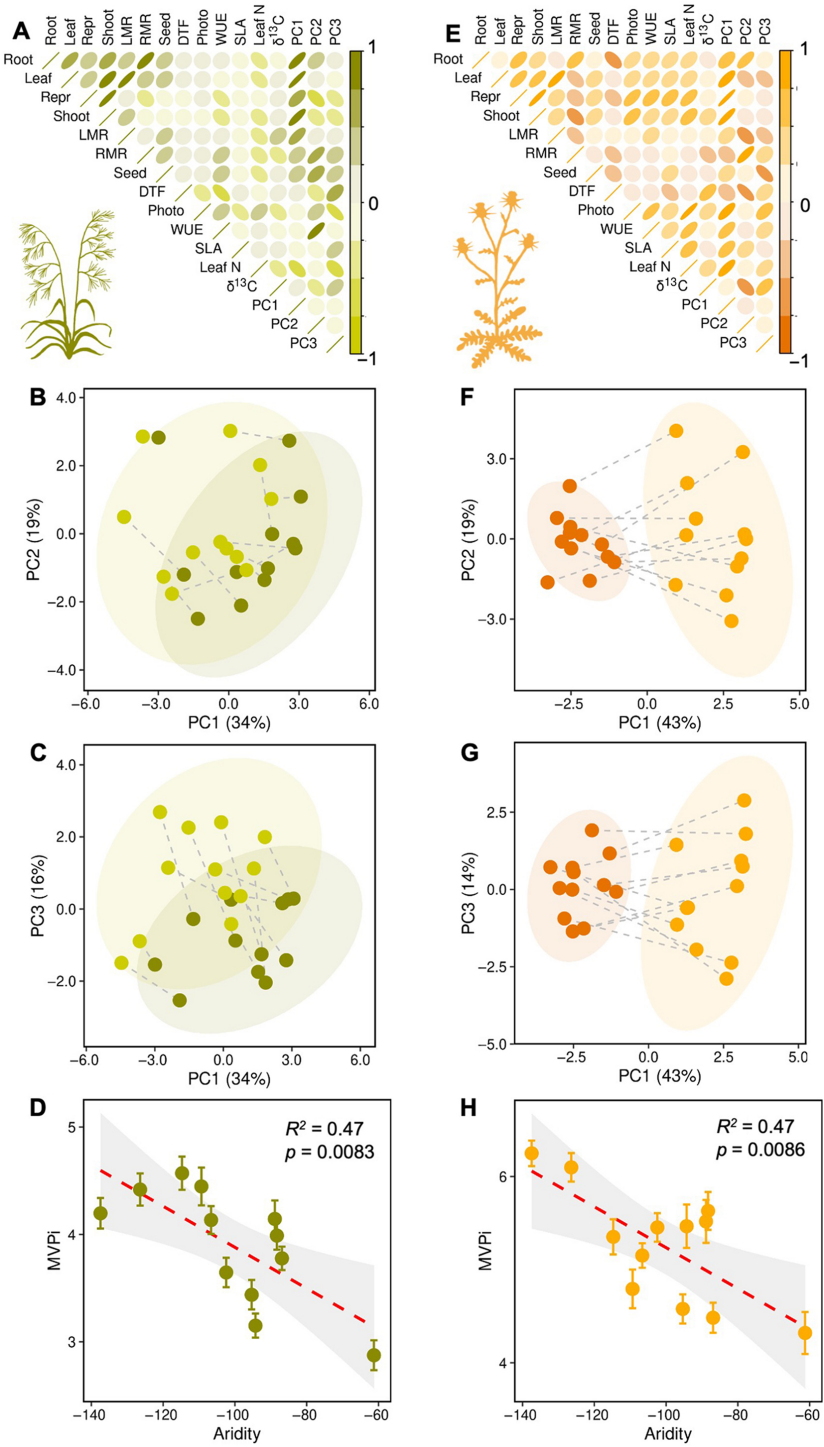
Results of principal components analysis (PCA) of plant traits for (A–C) *Bromus* and (E–G) *Centaurea*, including (A, E) correlations among traits and principal components, and (B, C, F, G) ordinations of population means under high (dark circles) and low (light circles) N among the first three principal components, with lines connecting means of the same population. Traits included were leaf, shoot, root, and reproductive biomass, leaf mass ratio (LMR), root mass ratio (RMR), mean seed mass, photosynthetic rate, water use efficiency (WUE), days to flowering (DTF), percent leaf N, and carbon isotope signatures. Also shown are results of linear regressions between multivariate plasticity (MVPi) and aridity of source populations for (D) *Bromus* and (H) *Centaurea*. Less negative aridity index values indicate more arid conditions. SLA, specific leaf area. Illustration credit: Justin M. Valliere.

We found substantial population‐level differences in overall plant plasticity using a multivariate approach (Pennacchi et al., 2021), and these responses were significantly influenced by climate variables (Table 2, Figure 5). PCAs of populations in trait space illustrated shifts in plant performance due to N availability, with these shifts driven by multiple plant traits. For both species, a large proportion of variance was explained by measures of plant growth, which were highly correlated with the first principal component (Figure 5A,E). In *Bromus*, the second principal component was correlated with several functional traits including RMR, seed mass, and WUE (Figure 5A), but while populations varied substantially in these traits, they did not show consistent changes due to N addition on the second principal component (Figure 5B). *Bromus* populations showed similar shifts along the third principal component in response to N addition, which was most strongly correlated with DTF and photosynthetic rate, and to a lesser extent other physiological traits (Figure 5A,C). Populations of *Centaurea* showed a more marked separation in trait space due to N addition on the first principal component, which was correlated with plant biomass, photosynthetic rate, WUE, SLA, and foliar N (Figure 5F). Populations also differed in placement along the second and third principal components, but these differences appeared unrelated to N addition (Figure 5F,G). The magnitude of overall trait shifts due to N addition (i.e., multivariate plasticity) varied across populations of both species. Moreover, these differences in overall trait plasticity were driven by the climate experienced by source populations; increasing aridity and lower precipitation were associated with reduced overall phenotypic plasticity in both species (Table 2, Figure 5D,H). For *Bromus*, greater plasticity was also positively associated with interannual rainfall variability (Table 2).

## DISCUSSION

We provide strong evidence that two widespread invasives exhibit population‐level differences in functional traits and levels of trait plasticity in response to soil N availability. Consistent with our hypotheses and previous work, we found that several morphological, physiological, and phenological traits were correlated with precipitation and aridity across populations. Given that these species have been present in California for centuries (Hendry, 1931) and experienced decades of elevated N deposition in some areas (Egerton‐Warburton et al., 2001), we also expected that some traits would be related to rates of N deposition experienced by source populations. However, we found no evidence that populations have experienced local adaptation in response to chronic N deposition, and instead the observed differences in trait values were largely explained by aridity. Populations from more arid sites exhibited lower trait plasticity in response to N addition, and levels of plasticity were explained by climate but not N deposition. While N deposition undoubtedly contributes to the success of these species throughout the region (Valliere et al., 2017, 2020), climate stress appears to shape population‐level trait differences, and these climate‐driven responses influence how these species respond to elevated N availability.

### Intraspecific trait variation is explained by climate not nitrogen deposition

Both resource availability and aridity might be expected to favor distinct suites of plant functional traits (Chapin et al., 1993; Tilman & Lehman, 2001). We observed substantial intraspecific variation in plant performance across populations of both species, but mean trait values showed few and relatively minor relationships with site‐level environmental conditions. Notably, only a single trait evaluated—photosynthetic rate of *Bromus* populations—showed a relationship with rates of N deposition; under low N availability, populations from high deposition sites exhibited reduced photosynthetic rates compared to those from low deposition sites. This could indicate that populations that have experienced decades of chronic N deposition are more reliant on elevated soil N for physiological functioning, or conversely, that populations from more N‐limited environments have adapted to operate at lower levels of soil N availability. However, we found no evidence that this lower photosynthetic capacity resulted in reduced plant growth or reproductive output in high N deposition populations.

In contrast, several functional traits were associated with aridity across populations. In *Bromus*, populations from more arid sites exhibited more rapid flowering, higher photosynthetic capacity, and greater WUE. This suggests that populations may have undergone selection for traits associated with both drought escape as well as drought tolerance (Welles & Funk, 2021). Previous work with another invasive *Bromus* species found that climate has driven similar ecotypic differentiation in phenology, with more arid populations exhibiting more rapid seed set than those from more mesic sites (Rice et al., 1992). In *Centaurea*, aridity had a similar effect on phenology, and we also observed climate‐driven differences in biomass allocation. Under high N availability, aridity was positively associated with root biomass and RMR and negatively associated with shoot biomass and LMR across populations. These results illustrate fundamentally different strategies for biomass allocation under high resource availability that are consistent with the levels of climate stress experienced by these populations. Previous studies have reported similar shifts in biomass allocation among invasive plant populations across aridity gradients (Lakoba & Barney, 2020; Welles & Funk, 2021).

### Climatic constraints on phenotypic plasticity under nitrogen deposition

Plants evolve in the context of complex and interactive environmental conditions, and the evolution of phenotypic plasticity in response to resource availability may be limited by environmental stress (Huang et al., 2015; Valladares et al., 2007). In this study, we found that aridity plays an important role in shaping trait plasticity. Increased climate stress at hotter and drier sites likely exerts a strong selective pressure on plant populations, and this could constrain plastic responses in several ways. First, earlier flowering in populations from more arid sites suggests that rapid development may restrict flexibility in growth and trait expression (Dyer et al., 2012). Second, while greater plasticity may be advantageous under some resource conditions, it may be maladaptive under more severe levels of climate stress (Huang et al., 2015; Van Kleunen & Fischer, 2005). For example, plants that allocate greater biomass to shoots or leaves may be more susceptible to drought events. We propose that observed differences in phenotypic plasticity are the result of climate‐driven, phenological constraints as well as selection against traits that could exacerbate water stress during periods of drought.

Although aridity appeared to constrain plasticity in both invasive species, differences in trait responses likely reflect their contrasting drought‐coping strategies. Annuals in water‐limited environments are typically characterized as drought escapists, but such species actually exhibit a diversity of traits and water‐use strategies (Rosenthal et al., 2010). *Bromus* is a winter annual that grows during the cooler winter months and flowers in spring. *Centaurea*, on the other hand, persists much later into summer. Thus, despite both being annuals, these species represent drought escape (i.e., avoiding drought through a more rapid lifecycle) and drought‐tolerant ecological strategies (i.e., avoiding dehydration through traits that increase water access or reduce water loss), respectively (Volaire, 2018).

In *Bromus*, the influence of climate on trait plasticity was largely driven by precipitation; populations that received more annual rainfall on average exhibited greater plasticity in shoot growth, reproductive output, and flowering phenology. Populations of *Bromus* from less arid sites also responded to N addition by increasing allocation to shoots, not roots. Given its earlier phenology, it is likely that winter rainfall is an important environmental cue driving selection in *Bromus*, and high nitrogen availability may reinforce traits that facilitate drought escape, such as increased shoot growth and more rapid reproduction.

In the late‐season forb *Centaurea*, we found that higher temperatures may also limit plasticity of shoot growth, while temperature had no effect on N responses in *Bromus*. *Centaurea* grows much later into the summer, and therefore elevated temperatures contribute to drought stress in a way not experienced by winter‐active species. We also observed key differences in how populations shifted biomass allocation in response to N availability in *Centaurea*. Populations from more arid sites responded to N addition by investing in greater root biomass, while populations from less arid sites increased allocation to leaves. These differences suggest distinct growth strategies and ecophysiological trade‐offs that may have evolved in response to local climatic conditions. Greater investment in leaves would allow plants to maximize carbon gain under favorable resource conditions, such as at more coastal and higher elevation sites where plants experience less severe water stress. However, this strategy would be much riskier under increasing aridity, which likely explains the opposite response observed in populations from more arid sites. In contrast, plasticity in biomass allocation and root growth was unrelated to climate variables in *Bromus*. This further illustrates that aridity may have different evolutionary impacts on species depending on their particular drought coping strategies.

### Lack of local adaptation in response to nitrogen deposition?

Given the dramatic biotic and abiotic environmental changes that N deposition may induce, why didn't we detect population‐level trait differences related to N deposition? While empirical evidence is extremely limited, chronic N deposition could be expected to lead to adaptive changes in functional traits (Tilman & Lehman, 2001). Two of the very few studies exploring the evolutionary consequences of N deposition on plant populations found evidence of genetically based differences in growth, phenology, and N use between plants from high and low deposition sites (Vergeer et al., 2008; Wedlich et al., 2016). Our results stand in contrast to this work; we found no evidence that intraspecific trait variation was related to exposure to N deposition. These two studies were conducted in Northwestern Europe where water availability plays a comparatively minor role in limiting plant growth (Vergeer et al., 2008; Wedlich et al., 2016), which may explain why aridity and not N deposition had an overwhelming effect on trait expression in our study. Our results are more consistent with those of Nguyen et al. (2016), who found evidence of rapid evolution of traits in two invasive grass species to experimental drought but not N addition. Unlike rainfall, soil N availability at a given site is relatively constant over time, and this lack of temporal variability likely explains why levels of trait plasticity were unrelated to rates of N deposition (Botero et al., 2015). The aforementioned studies from Europe also found evidence of reduced phenotypic plasticity in populations from high deposition sites (Vergeer et al., 2008; Wedlich et al., 2016), but we detected no such pattern in these species across a variety of traits.

Collectively, these findings support the idea that climate stress may exert an overriding constraint on adaptive responses in invasive species in dryland ecosystems. However, there may be alternative explanations for a lack of adaptive responses to N deposition. First, there may have been insufficient time for populations to evolve in response to soil N availability, or variability in N deposition across sites may not have been strong enough to detect trait differences in this study. This seems unlikely, as these are annual plant species that have experienced many generations (i.e., decades) of highly elevated N deposition at some sites (Egerton‐Warburton et al., 2001). Further, our previous experimental and observational work in this system has found that even moderate levels of N deposition can result in highly elevated soil N and substantial impacts on invasive plant performance, community structure, and competitive interactions (Valliere et al., 2017, 2020, 2022). Second, it could be that these species lack the genetic variation necessary to elicit evolutionary changes in response to N deposition. Previous work in *Centaurea* and other invasive species of *Bromus* has found important biogeographic differences in N responses among native and introduced populations (He et al., 2011; Moroney et al., 2013). For example, Moroney et al. (2013) found that invasive populations of *Centaurea* were more responsive to N fertilization than populations from the native range in Spain. However, this does not necessarily mean that the populations in our study possessed sufficient genetic variation for selection to act upon. Nevertheless, based on our findings and previous work (Nguyen et al., 2016) we believe that constraints imposed by climate adaptation are the most likely explanation for the lack of adaptation to N deposition.

### Potential limitations and future directions

Our experiment was conducted at a single common garden, which allowed us to detect clear population‐level differences in traits and trait plasticity in both species. However, rigorous tests of local adaptation require reciprocal transplant experiments to rule out site‐specific effects and assess if such trait differences truly confer home‐site advantages (Johnson et al., 2022; Leimu & Fischer, 2008). While we cannot fully exclude the influence of stochastic factors at our common garden site, the strong and consistent climate–trait relationships we observed lend compelling support to the idea that these population‐level differences reflect adaptive responses to aridity (which in turn, influence responses to N). Additionally, this study was conducted using field‐collected seed produced by plants that experienced variable environmental conditions; therefore, maternal effects could have impacted the patterns observed. However, we observed no influence of maternal seed mass on plant growth. It is still possible that observed responses were influenced by maternal effects, but these are often transitory and typically influence early life history traits (Bischoff & Müller‐Schärer, 2010), whereas we grew plants to reproductive maturity. For example, in *Centaurea maculosa* (a congeneric of one study species), Weiner et al. (1997) found a weak influence of maternal effects on seedling performance, and these disappeared after 8 weeks. It should also be noted that we did not manipulate water availability, which could have important interactive effects on trait expression in conjunction with N availability (Valliere, 2019), and this is an important avenue for future research. Previous work has shown that these species succeed over natives under both drought and N deposition due to greater growth and reproductive output (Valliere et al., 2019) as well as phenological and physiological advantages (Valliere et al., 2022). Comparisons of adaptive responses between native and invasive species in response to these global change drivers will be useful for evaluating the role of rapid evolution in driving these ecological outcomes.

## CONCLUSIONS

Evolutionary responses to novel environmental conditions may play an important role in the invasion process (Moran & Alexander, 2014; Whitney & Gabler, 2008). Our work demonstrates that N addition may indeed enhance the performance of invasive plant species, but local adaptation to aridity might constrain the capacity of invasives to respond to elevated N deposition. Therefore, while N deposition exacerbates the negative ecological consequences of invasive plants (Cox et al., 2014; Fenn et al., 2010; Valliere et al., 2020), climate stress may limit the evolutionary potential of plant invaders in dryland ecosystems subject to N deposition. The species evaluated here are already widespread throughout the region, but rapid adaptation to climatic conditions could have facilitated past range expansions and could also contribute to future spread into new environments (Colautti & Barrett, 2013), further endangering native biodiversity under ongoing environmental change.

## CONFLICT OF INTEREST STATEMENT

The authors declare no conflicts of interest.

## Supporting information


Appendix S1.

## Data Availability

Data (Valliere et al., 2025) are available in Dryad at https://doi.org/10.5061/dryad.r4xgxd2r3.
