# Supplementary material for: Evidence of local adaptation to aridity but not nitrogen deposition in invasive annuals
Source: Ecology. 2025 Jul 29;106(7):e70172. doi: 10.1002/ecy.70172 (PMC12305752; doi:10.1002/ecy.70172)
Supplement: Supplementary file 1 — Appendix S1. [file ECY-106-e70172-s001.pdf]

# Evidence of local adaptation to aridity but not nitrogen deposition in invasive annuals

Justin M. Valliere, Mayra J. Hernández, M. Rasoul Sharifi, and Philip W. Rundel

*Ecology*

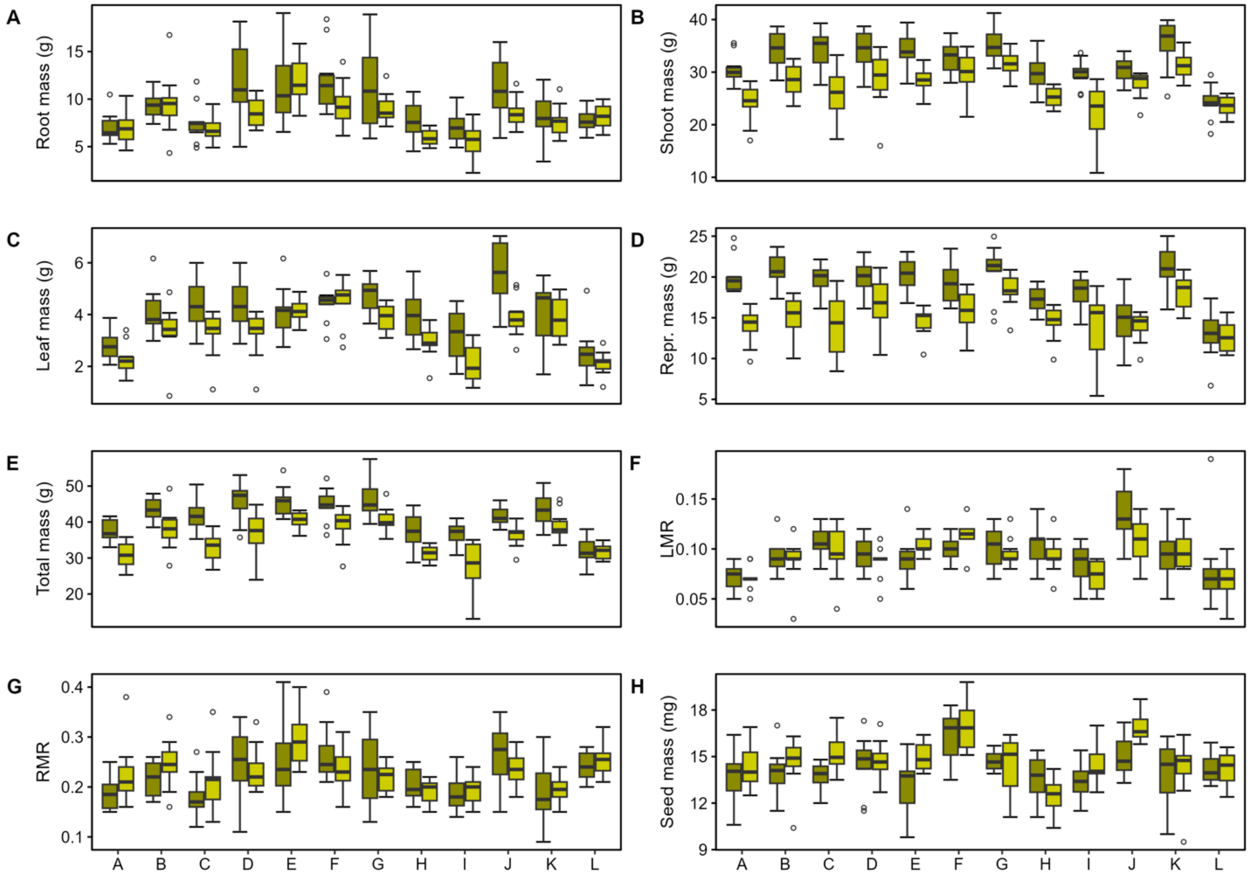


**Figure S1**. Boxplots of plant traits of twelve different source populations of *Bromus diandrus* grown under high (dark green) and low (light green) N availability in a common garden experiment from seed collected from throughout southern California. Sites are ordered from left to right according to increasing aridity. Traits include measures of plant growth (A-E; root, shoot, leaf, reproductive, and total biomass), allocation (F-G; leaf mass ratio [LMR] and root mass ratio [RMR]), and mean seed mass (H). Boxplots display minimum, maximum, and median values and interquartile range.

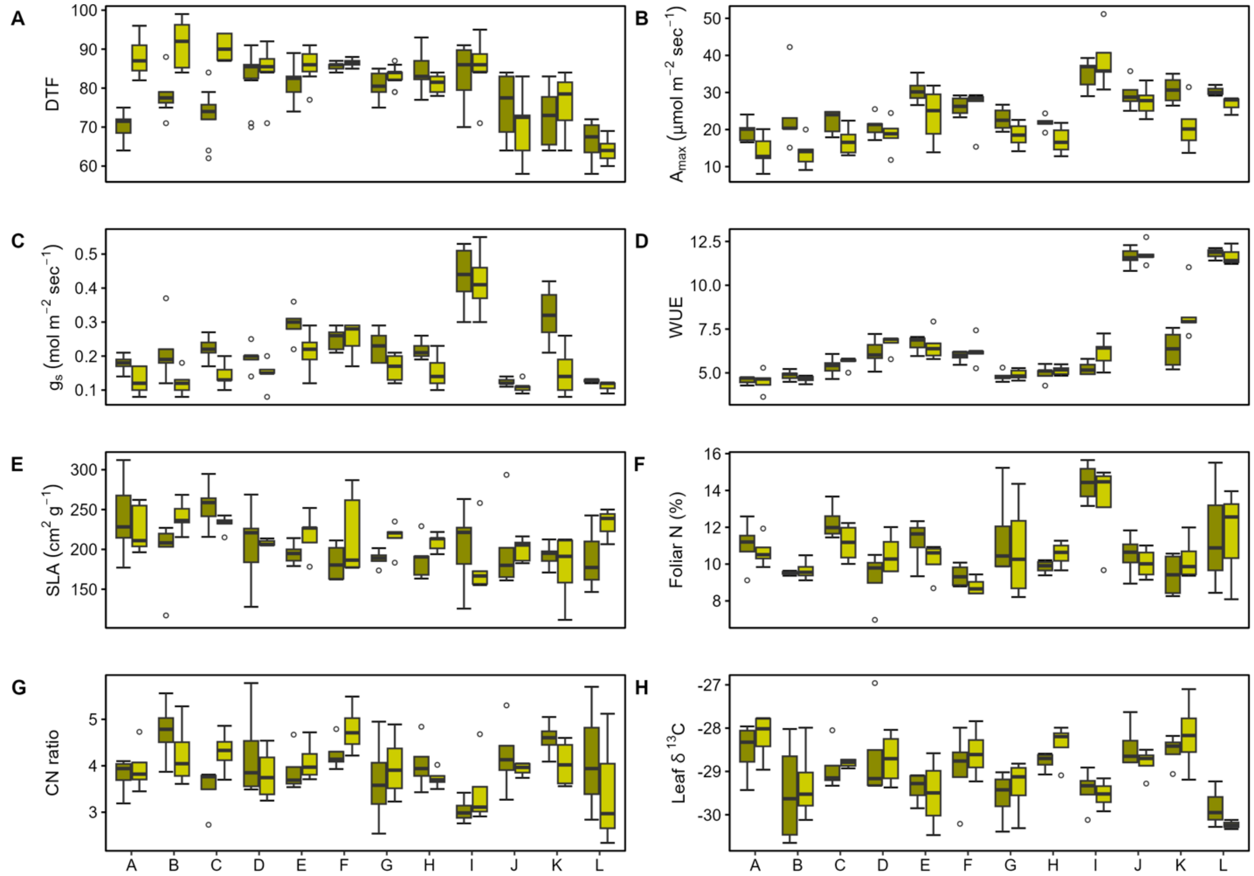


**Figure S2**. Boxplots of plant traits of twelve different source populations of *Bromus diandrus* grown under high (dark green) and low (light green) N availability in a common garden experiment from seed collected throughout southern California. Sites are ordered from left to right according to increasing aridity. Traits include days to flowering (A; DTF), photosynthetic capacity (B; $A_{max}$), stomatal conductance (C; $g_s$), instantaneous water-use efficiency (D; WUE), specific leaf area (E; SLA), foliar N concentration (F), leaf CN ratio (G), and leaf carbon isotope ratio (H). Boxplots display minimum, maximum, and median values and interquartile range.

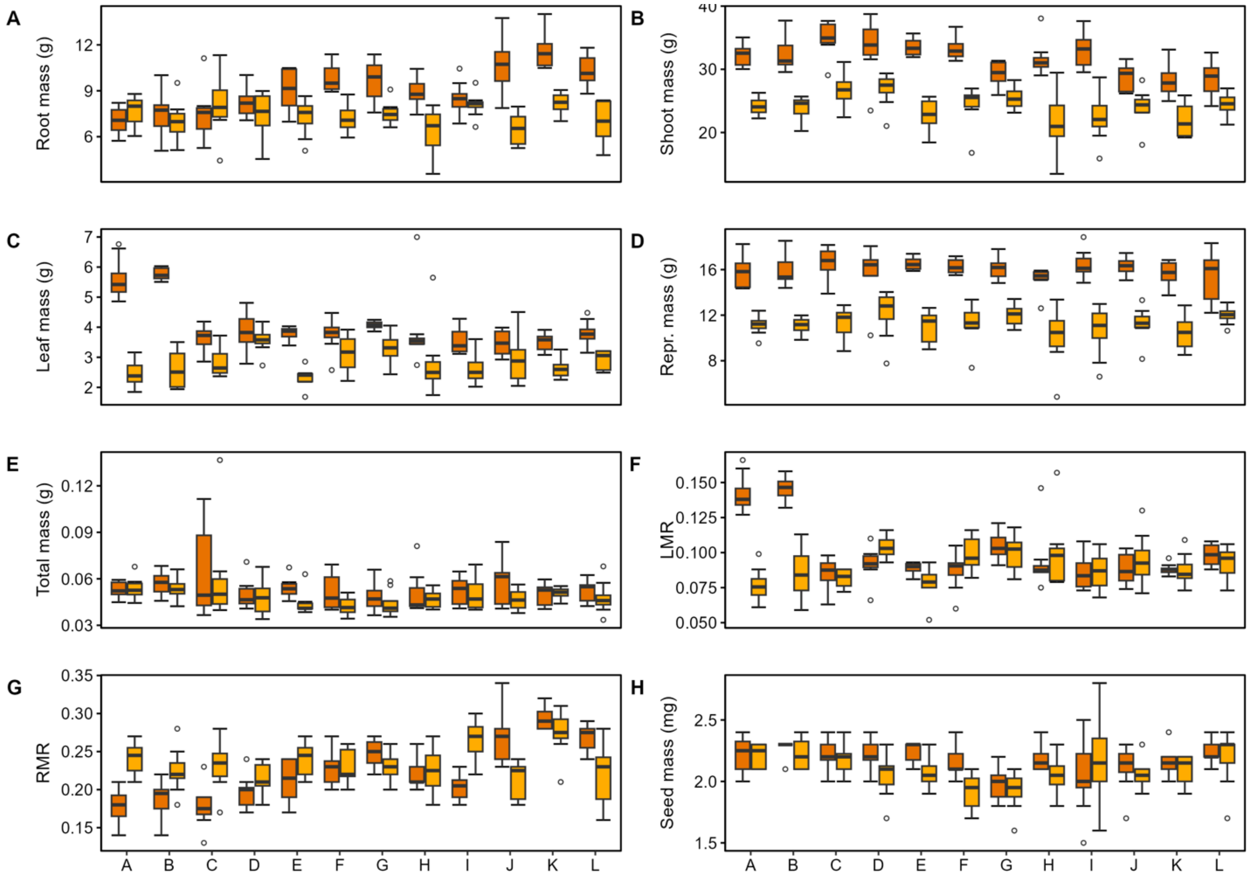


**Figure S3**. Boxplots of plant traits of twelve different source populations of *Centaurea melitensis* grown under high (dark green) and low (light green) N availability in a common garden experiment from seed collected throughout southern California. Sites are ordered from left to right according to increasing aridity. Traits include measures of plant growth (A-E; root, shoot, leaf, reproductive, and total biomass), allocation (F-G; leaf mass ratio [LMR] and root mass ratio [RMR]), and mean seed mass (H). Boxplots display minimum, maximum, and median values and interquartile range.

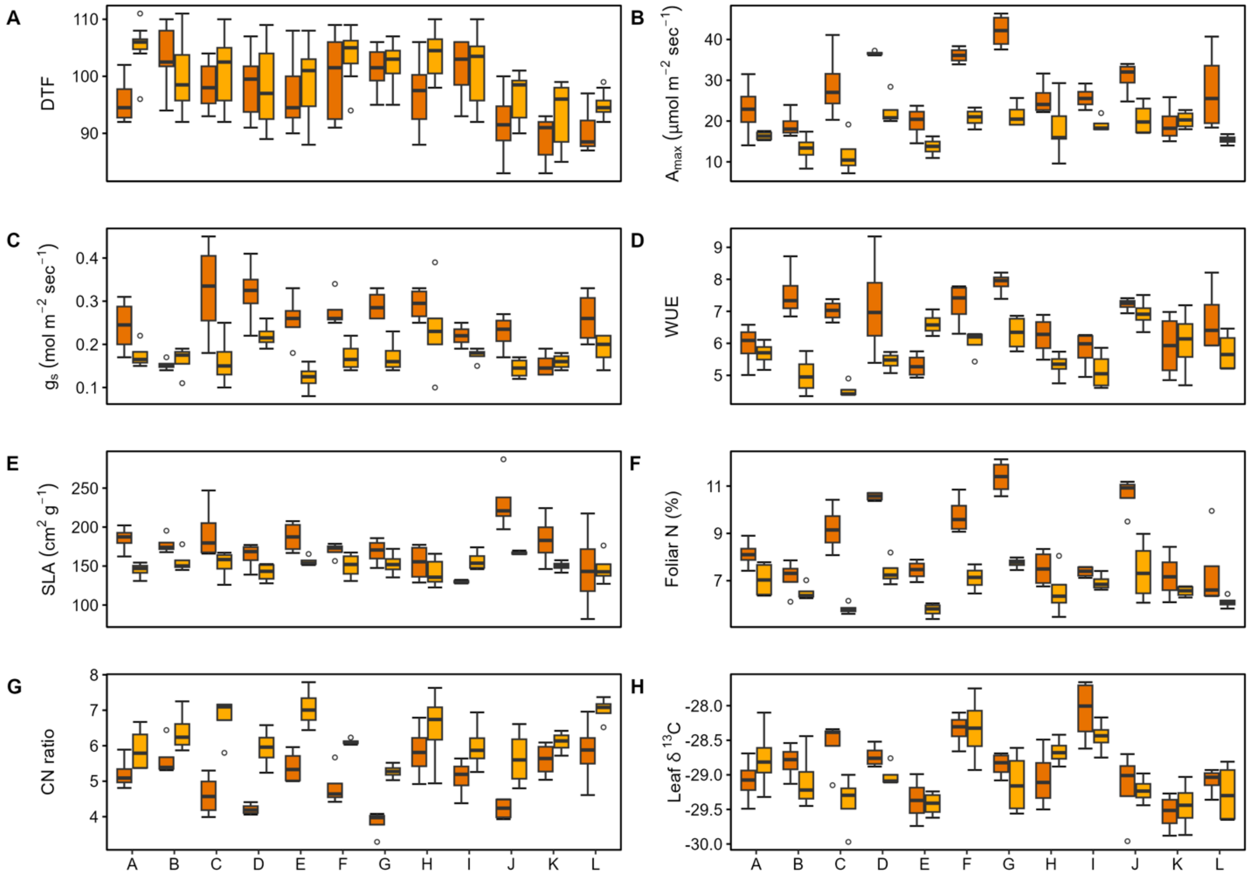


**Figure S4**. Boxplots of plant traits of twelve different source populations of *Centaurea melitensis* grown under high (dark green) and low (light green) N availability in a common garden experiment from seed collected throughout southern California. Sites are ordered from left to right according to increasing aridity. Traits include days to flowering (A; DTF), photosynthetic capacity (B; $A_{max}$), stomatal conductance (C; $g_s$), instantaneous water-use efficiency (D; WUE), specific leaf area (E; SLA), foliar N concentration (F), leaf CN ratio (G), and leaf carbon isotope ratio (H). Boxplots display minimum, maximum, and median values and interquartile range.

**Table S1**. Site information for each source population of *Bromus diandrus* and *Centaurea melitensis* used in the common garden experiment, including latitude, longitude, and elevation. Also included are modeled rates of N deposition for each site from several sources, including from a model available from the University of California Riverside for 2002 (Tonnesen et al. 2007, Fenn et al. 2010), which was used for initial site selection, and more recent iterations of the Community Multiscale Air Quality (CMAQ) Modeling System for 2002 and 2017 (Benish et al. 2022). We used mean N deposition rates for this time period for our statistical analyses, as this best reflects long-term patterns of exogenous N inputs across sites. Also shown are climate variables used for evaluating relationships between plant traits and trait plasticity and climate factors, including mean annual precipitation, mean maximum annual temperatures, and aridity for each site.

| | | | | N deposition (kg $ha^{-1}$ $yr^{-1}$) | | | | Climate variables | | |
|---|---|---|---|---|---|---|---|---|---|---|
| Site | Latitude | Longitude | Elev. (m) | UCR 2002 | CMAQ 2002 | CMAQ 2017 | Mean 2002-17 | Prec. (mm) | T max (°C) | Aridity index |
| Leo Carrillo | 34.06018 | -118.94035 | 221 | 1.3 | 8.6 | 6.0 | 7.7 | 420 | 22.9 | -94 |
| Satwiwa | 34.14858 | -118.95576 | 286 | 8.8 | 9.7 | 6.4 | 8.2 | 458 | 22.7 | -107 |
| Stunt Ranch | 34.09395 | -118.65514 | 424 | 13.3 | 13.2 | 8.9 | 11.7 | 562 | 24.4 | -137 |
| Franklin Canyon | 34.12432 | -118.40886 | 310 | 20.1 | 13.3 | 8.4 | 11.1 | 506 | 25.5 | -126 |
| Palos Verdes | 33.75308 | -118.38713 | 236 | 11.7 | 4.5 | 2.9 | 4.1 | 365 | 21.7 | -89 |
| Baldwin Hills | 34.01420 | -118.36962 | 128 | 19.9 | 13.7 | 9.3 | 12.2 | 399 | 22.8 | -102 |
| Griffith Park | 34.12751 | -118.29872 | 347 | 21.8 | 14.7 | 10.0 | 13.0 | 481 | 25.3 | -115 |
| Powder Canyon | 33.97212 | -117.92889 | 299 | 52.3 | 12.6 | 8.8 | 11.1 | 431 | 25.3 | -109 |
| Jurupa Hills | 34.03822 | -117.47738 | 276 | 22.0 | 16.4 | 9.9 | 13.5 | 334 | 26.1 | -87 |
| Starr Ranch | 33.62697 | -117.55110 | 355 | 8.0 | 9.3 | 5.8 | 7.9 | 418 | 24.3 | -95 |
| Box Springs | 33.96573 | -117.32223 | 373 | 14.7 | 12.4 | 7.2 | 9.9 | 263 | 26.0 | -61 |
| Lake Skinner | 33.58820 | -117.02795 | 471 | 8.7 | 6.0 | 3.4 | 4.9 | 358 | 25.4 | -88 |

**Table S2**. Results of two-way analysis of variance (ANOVA) models evaluating the effect of source population, N availability, and their interaction on plant growth metrics and functional traits for *Bromus diandrus*, including degrees of freedom, F values, and p values. Significant models are bolded.

| | Population | | | Nitrogen | | | Population x Nitrogen | | |
|---|---|---|---|---|---|---|---|---|---|
| Variable | df | F | p | df | F | p | df | F | p |
| Root mass | **11, 216** | **11.05** | **<0.0001** | **1, 216** | **11.28** | **0.0009** | 11, 216 | 1.05 | 0.3995 |
| Shoot | **11, 216** | **14.24** | **<0.0001** | **1, 216** | **114.11** | **<0.0001** | **11, 216** | **1.94** | **0.0364** |
| Leaf mass | **11, 216** | **16.73** | **<0.0001** | **1, 216** | **42.43** | **<0.0001** | **11, 216** | **1.94** | **0.0360** |
| Repr. mass | **11, 216** | **12.21** | **<0.0001** | **1, 216** | **122.20** | **<0.0001** | **11, 216** | **2.84** | **0.0017** |
| Total mass | **11, 216** | **19.17** | **<0.0001** | **1, 216** | **114.79** | **<0.0001** | 11, 216 | 1.49 | 0.1371 |
| LMR | **11, 216** | **9.02** | **<0.0001** | 1, 216 | 3.51 | 0.0623 | 11, 216 | 1.63 | 0.0913 |
| RMR | **11, 216** | **6.32** | **<0.0001** | 1, 216 | 2.13 | 0.1455 | 11, 216 | 1.13 | 0.3384 |
| Seed mass | **11, 216** | **8.53** | **<0.0001** | **1, 216** | **10.39** | **0.0015** | **11, 216** | **1.90** | **0.0405** |
| DTF | **11, 216** | **23.29** | **<0.0001** | **1, 216** | **50.25** | **<0.0001** | **11, 216** | **12.23** | **<0.0001** |
| $A_{max}$ | **11, 91** | **14.27** | **<0.0001** | **1, 91** | **23.04** | **<0.0001** | 11, 91 | 1.63 | 0.1038 |
| $g_s$ | **11, 91** | **17.24** | **<0.0001** | **1, 91** | **45.48** | **<0.0001** | 11, 91 | 1.78 | 0.0686 |
| WUE | **11, 91** | **46.44** | **<0.0001** | **1, 91** | **4.22** | **0.0427** | 11, 91 | 1.44 | 0.1680 |
| SLA | **11, 91** | **2.37** | **0.0124** | 1, 91 | 2.03 | 0.1573 | 11, 91 | 1.30 | 0.2380 |
| Foliar N | **11, 70** | **4.59** | **<0.0001** | 1, 70 | 0.22 | 0.6403 | 11, 70 | 0.77 | 0.7723 |
| CN | **11, 70** | **1.98** | **0.0436** | 1, 70 | 0.01 | 0.9285 | 11, 70 | 0.96 | 0.4929 |
| $\delta^{13}C$ | **11, 70** | **4.88** | **<0.0001** | 1, 70 | 0.38 | 0.5381 | 11, 70 | 0.34 | 0.9730 |

**Table S3**. Results of two-way analysis of variance (ANOVA) models evaluating the effect of source population, N availability, and their interaction on plant growth metrics and functional traits for *Centaurea melitensis*, including degrees of freedom, F values, and p values. Significant models are bolded.

| | Population | | | Nitrogen | | | Population x Nitrogen | | |
|---|---|---|---|---|---|---|---|---|---|
| Variable | df | F | p | df | F | p | df | F | p |
| Root mass | **11, 168** | **4.73** | **<0.0001** | **1, 168** | **80.21** | **<0.0001** | **11, 168** | **5.83** | **<0.0001** |
| Shoot | **11, 168** | **5.45** | **<0.0001** | **1, 168** | **358.66** | **<0.0001** | **11, 168** | **3.13** | **0.0007** |
| Leaf mass | **11, 168** | **4.28** | **<0.0001** | **1, 168** | **203.38** | **<0.0001** | **11, 168** | **7.87** | **<0.0001** |
| Repr. mass | 11, 168 | 1.11 | 0.3602 | **1, 168** | **483.06** | **<0.0001** | 11, 168 | 0.97 | 0.4757 |
| Total mass | **11, 168** | **2.28** | **0.0130** | **1, 168** | **338.16** | **<0.0001** | 11, 168 | 1.62 | 0.0976 |
| LMR | **11, 168** | **5.40** | **<0.0001** | **1, 168** | **17.77** | **<0.0001** | **11, 168** | **10.69** | **<0.0001** |
| RMR | **11, 168** | **12.03** | **<0.0001** | **1, 168** | **8.19** | **0.0048** | **11, 168** | **9.89** | **<0.0001** |
| Seed mass | **11, 168** | **3.86** | **<0.0001** | **1, 168** | **8.71** | **0.0036** | 11, 168 | 1.40 | 0.1749 |
| DTF | **11, 168** | **8.06** | **<0.0001** | **1, 168** | **15.37** | **0.0001** | **11, 168** | **1.85** | **0.0498** |
| $A_{max}$ | **11, 73** | **7.81** | **<0.0001** | **1, 73** | **95.51** | **<0.0001** | **11, 73** | **2.33** | **0.0160** |
| $g_s$ | **11, 73** | **4.00** | **0.0001** | **1, 73** | **51.88** | **<0.0001** | **11, 73** | **2.10** | **0.0311** |
| WUE | **11, 73** | **4.61** | **<0.0001** | **1, 73** | **46.56** | **<0.0001** | **11, 73** | **5.15** | **<0.0001** |
| SLA | **11, 73** | **3.57** | **0.0005** | **1, 73** | **21.20** | **<0.0001** | **11, 73** | **2.03** | **0.0377** |
| Foliar N | **11, 72** | **10.50** | **<0.0001** | **1, 72** | **120.91** | **<0.0001** | **11, 72** | **2.89** | **0.0034** |
| CN | **11, 72** | **7.28** | **<0.0001** | **1, 72** | **99.47** | **<0.0001** | 11, 72 | 1.50 | 0.1489 |
| $\delta^{13}C$ | **11, 72** | **8.72** | **<0.0001** | 1, 72 | 3.00 | 0.0876 | 11, 72 | 1.72 | 0.0862 |

**Table S4**. Coefficients of determination ($R^2$) from simple linear regressions evaluating the effect of site-level N deposition rates and aridity on mean trait values across populations (n = 12) of *Bromus diandrus* and *Centaurea melitensis* under both low and high N availability. Asterisks denote levels of significance (*$p < 0.05$; **$p < 0.01$; ***$p < 0.001$), with significant regressions shown in bold.

| | *Bromus diandrus* | | | | *Centaurea melitensis* | | | |
|---|---|---|---|---|---|---|---|---|
| | N dep | | Aridity | | N dep | | Aridity | |
| Trait | Low N | High N | Low N | High N | Low N | High N | Low N | High N |
| Root mass | -0.09 | -0.09 | -0.09 | -0.10 | 0.05 | -0.08 | -0.06 | **0.58**** |
| Shoot mass | 0.02 | 0.04 | -0.09 | 0.20 | 0.02 | -0.07 | -0.03 | **0.34*** |
| Leaf mass | -0.06 | -0.08 | -0.10 | -0.10 | -0.10 | -0.02 | -0.05 | **0.45**** |
| Repr. mass | 0.01 | 0.13 | -0.09 | **0.42*** | -0.08 | -0.06 | -0.09 | -0.03 |
| Total mass | -0.01 | -0.04 | -0.09 | 0.07 | 0.09 | -0.08 | -0.01 | -0.04 |
| LMR | -0.09 | 0.02 | -0.10 | -0.06 | -0.07 | -0.04 | 0.11 | **0.33*** |
| RMR | -0.10 | -0.04 | -0.09 | -0.01 | -0.09 | -0.08 | -0.10 | **0.58**** |
| Seed mass | -0.10 | -0.07 | -0.10 | -0.08 | -0.09 | 0.25 | -0.05 | 0.04 |
| DTF | -0.02 | 0.03 | **0.65***** | -0.09 | -0.10 | -0.09 | **0.27*** | 0.19 |
| $A_{max}$ | **0.33*** | 0.13 | **0.33*** | **0.35*** | -0.04 | -0.09 | 0.01 | -0.06 |
| WUE | -0.04 | -0.02 | **0.49**** | **0.39*** | 0.01 | -0.08 | 0.01 | -0.10 |
| SLA | 0.04 | -0.03 | 0.08 | 0.22 | 0.18 | -0.09 | -0.08 | -0.01 |
| N | 0.10 | 0.19 | 0.02 | -0.06 | -0.01 | -0.09 | -0.10 | -0.10 |
| $\delta^{13}C$ | 0.06 | -0.05 | 0.14 | 0.01 | -0.04 | -0.07 | -0.09 | -0.09 |

## REFERENCES

Benish, S. E., J. O. Bash, K. M. Foley, K. W. Appel, C. Hogrefe, R. Gilliam, and G. Pouliot. 2022. Long-term regional trends of nitrogen and sulfur deposition in the United States from 2002 to 2017. Atmos. Chem. Phys. **22**:12749-12767.

Fenn, M. E., E. B. Allen, S. B. Weiss, S. Jovan, L. H. Geiser, G. S. Tonnesen, R. F. Johnson, L. E. Rao, B. S. Gimeno, F. Yuan, T. Meixner, and A. Bytnerowicz. 2010. Nitrogen critical loads and management alternatives for N-impacted ecosystems in California. J Environ Manage **91**:2404-2423.

Tonnesen, G., Z. Wang, M. Omary, and C. Chien. 2007. Assessment of nitrogen deposition: modeling and habitat assessment. California Energy Commission, PIER Energy-Related Environmental Research, Sacramento: www. energy. ca. gov/2006publications/CEC-500-2006-032 (Accessed December 2009).
